# Renal Abnormalities Caused by Canine Distemper Virus Infection in Terminal Patients

**DOI:** 10.3389/fvets.2022.822525

**Published:** 2022-03-08

**Authors:** Mayra de Lima e Silva, Gyl Eanes Barros Silva, Sofia Borin-Crivellenti, Alef Winter Oliveira Alvarenga, Marcela Aldrovani, Larissa Ayane do Nascimento Braz, Caroline Aoki, Aureo Evangelista Santana, Caio Santos Pennacchi, Leandro Zuccolotto Crivellenti

**Affiliations:** ^1^Animal Science Graduate Program, Veterinary Teaching Hospital of Universidade de Franca, São Paulo, Brazil; ^2^Department of Renal Pathology, Universidade Federal do Maranhão, São Luís, Brazil; ^3^Graduate Program in Veterinary Science (PPGCV), College of Veterinary Medicine (FAMEV), Universidade Federal de Uberlândia, Uberlândia, Brazil; ^4^Department of Clinical and Veterinary Surgery, School of Agricultural and Veterinary Sciences, Universidade Estadual Paulista (UNESP/FCAV), Jaboticabal, Brazil

**Keywords:** glomerulonephritis, glomerulosclerosis, proteinuria, tubular necrosis, *Paramyxoviridae*, microscopy

## Abstract

The aim of this study was to analyze the glomerular and tubular alterations in dogs with terminal distemper through light microscopy, immunofluorescence, and electron microscopy. Thirteen animals with a molecular diagnosis of distemper and neurological signs were selected. As a control group, 10 clinically healthy animals with no manifestations or signs of disease and with negative tests for *Ehrlichia* sp., *Anaplasma* sp., and *Babesia* sp. were included in this study. Renal tissue was evaluated by light microscopy, topochemistry for DNA/chromatin, and video image analysis to detect the nuclear phenotypes of the renal tubular epithelial cells (RTECs), immunofluorescence, and transmission electron microscopy. Results showed that dogs with distemper exhibited anemia, hypergammaglobulinemia, and proteinuria. Creatinine in the distemper group was lower compared to the control group (*p* = 0.0026), but there was no significant difference in relation to urea (*p* = 0.9876). Although this alteration may be due to the smaller muscle mass observed in animals with distemper, it probably is not of clinical importance. Glomerular and tubular lesions were confirmed by light microscopy in 84.6% of these animals. Additional findings in the animals with distemper included deposition of different classes of immunoglobulins, particularly IgM in 92.3% of the cases, fibrinogen deposition in 69.2% of the cases as assessed by immunofluorescence, alterations in the nuclear phenotypes of the RTEC characterized by condensation of chromatin, loss of DNA and reduction in the nuclear shape, and the presence of subendothelial and mesangial electron-dense deposits. These findings confirm the existence of renal alterations related to terminal distemper.

## Introduction

Morbillivirus is a genus of single-stranded, negative-sense RNA viruses that belong to the *Paramyxoviridae* family (1–3). This genus, which has a wide range of hosts, develops in both humans and animals (4), with cases of infection described in canids, bovids, equines, primates, small ruminants, felids, aquatic animals, and birds, among other taxa (1, 2, 4). Consequently, morbilliviruses have great relevance for public health, livestock, and wildlife conservation (5).

The clinical signs of the diseases caused by morbilliviruses are very similar, ranging from mild to severe, and may lead to death (3). Multisystemic alterations occur, especially with cutaneous, respiratory, gastrointestinal, and neurological lesions, despite the fact this group of viruses reportedly causes renal damage (6).

Renal alterations described in measles and feline morbillivirus infection include interstitial nephritis and acute tubular necrosis (7–9). Changes in the tubules and epithelia of the renal pelvis have been reported in animals infected with the small ruminant plague virus (10). Renal necrosis was observed in horses and humans diagnosed with a new type of morbillivirus (11), and in birds infected with paramyxovirus type 1 (12).

The number of species affected by the virus that causes canine distemper has been increasing; this virus is highly neurovirulent, especially to young animals, and is associated with a high mortality rate (13). However, the urinary system is poorly investigated in cases of canine distemper, with renal cell lesions described only in cases of experimental infection (14). Moreover, alterations of the tubular and glomerular components of the kidney have not yet been described in this disease. Considering that distemper is a serious disease that affects several organs, including the urinary system, a more in-depth study is proposed to evaluate the renal alterations in infected animals.

## Materials and Methods

The use of the animals followed an experimental protocol previously approved by the Animal Care and Use Committee (Comissão de Ética no Uso de Animais, CEUA) of Universidade de Franca UNIFRAN-SP (protocol # 067/15) and FCAV-UNESP (protocol 006353/12 – May 24, 2012.

### Animals

Two experimental groups, homogeneous for weight and age (*p* > 0.05) were used in this study. The animals included in these groups were selected from those routinely examined at the Small Animal Clinical Medicine services (Serviços de Clínica Médica de Pequenos Animais) of the Veterinary Hospitals of Universidade de Franca (UNIFRAN-SP) and FCAV/UNESP, Jaboticabal campus.

Group I (control) consisted of 10 clinically healthy dogs of various breeds, with no known disease. The weight of the animals of the control group was between 5 and 18 kg (10.1 ± 4.2 kg). These animals were between 3 and 8 months of age (6.5 ± 1.9 months), had negative PCR tests for *Ehrlichia* sp., *Babesia* sp. and *Anaplasma* sp., and negative serology for *E. canis* and *Leptospira* sp. The health status was assessed by means of physical examination, hematological and biochemical evaluations of serum samples, and urine tests.

Group II (distemper) consisted of 13 dogs of various breeds, with ages between 4 months and 2 years (11.1 ± 6.4 months), and weight between 2 and 20 kg (11 ± 12.7 kg). The animals in this group exhibited neurological signs of distemper, tested positive for the distemper virus as assessed by real-time PCR, were negative for *Ehrlichia* sp. as assessed by PCR, were negative for *Babesia* sp. and *Anaplasma* sp. as assessed by semi-nested PCR, and had also negative serology for *Leptospira* sp. (IgM and IgG), toxoplasmosis, and neosporosis.

Urine and blood samples were collected from the animals of both groups for complete blood count (PocH−100iv Diff), biochemical profiling (Labquest—Labtest Diagnóstica—Belo Horizonte—MG—Brazil), urinalysis, and determination of urinary protein/creatinine (UPC) ratios.

Both serum and urine were evaluated using SDS-PAGE. Serum IgG and IgA were visualized after staining with Coomassie Blue (Sigma-Aldrich). After centrifugation, the 10-mL urine supernatants were transferred to separate tubes for analysis of biochemical and urinary proteins. The samples were fractionated by SDS-PAGE using 10.4% polyacrylamide with molecular-weight size markers (α-lactalbumin: 14.2 kDa; soybean trypsin inhibitor: 20.0 kDa; bovine pancreas trypsinogen: 24 kDa; carbonic anhydrase from bovine erythrocytes: 29 kDa; rabbit muscle glyceraldehyde-3-phosphate dehydrogenase: 36 kDa; ovalbumin: 45 kDa; albumin: 66 kDa; phosphorylase b: 97 kDa; E. coli P-galactosidase: 116 kDa; rabbit muscle myosin: 205 kDa). All markers were from SigmaMarker, USA.

### Biopsy Material Collection

Group I (control): renal biopsies were performed after ovariohysterectomy using a semi-automatic device with an 18-, 16-, or 14-gauge Tru-cut^®^ cutting needle. Samples were collected from the surface of the renal cortex of the left kidney.

Group II (distemper): animals were euthanized and the samples collected by dissection of the kidney using a scalpel under direct visualization.

### Biopsy Sample Processing

The samples were sectioned into three pieces and these pieces immersed separately in alcoholic Bouin's solution, Michel's solution, and glutaraldehyde, for evaluation by light microscopy (LM) and video image analysis to describe nuclear phenotypes, immunofluorescence (IF), and transmission electron microscopy (TEM).

For LM, the samples were processed for routine inclusion in paraffin and cut at a thickness of 2–3 μm. Hematoxylin and eosin (HE), periodic acid-Schiff (PAS), Jones methenamine silver (JMS), or Masson's trichrome (TRI) were used to stain the sections. Samples suspected to contain amyloid deposition were cut at a thickness of 6 μm and stained with Congo Red.

To assess the nuclear phenotypes of the renal tubular epithelial cells (RTECs), the samples were included in paraffin and cut at a thickness of 6 μm. The sections were subjected to the Feulgen reaction, a topochemical method for the detection of DNA chromatin (15) consisting of acid hydrolysis (4 M HCl for 60 min at 25°C) and exposure of the hydrolyzed material to a reactive Schiff base (Merck) for 40 min in the dark. Feulgen-stained nuclei were evaluated under an Olympus BX53 light microscope (Tokyo, Japan) equipped with Plan Neofluar 40/0.75 and 100/0.75 objectives, condenser 0.90, and an interference bandpass filter to obtain monochromatic light of 546 nm (Edmund Optics, Barrington, NJ). With a high-resolution video camera (Olympus), images were transmitted to a computer in which they were digitized and analyzed using ImageJ software (National Institutes of Health, Bethesda, MD), as described previously (15). The video image parameters used to describe the nuclear phenotypes of the RTECs were the following: nuclear area (μm^2^) nuclear perimeter (μm); optical density (OD), which described the degree of chromatin condensation; standard deviation of the gray average in pixels per nuclei (SDtd), which described the contrast between the areas of greater- and lesser-condensed chromatin; and integrated optical density (IOD), which determined the DNA content in arbitrary units (AU). For each group, 500 cell nuclei were examined.

For IF, the sample was frozen in liquid nitrogen and cut at a thickness of 2–3 μm at −20°C in a cryostat. After incubation for 30 min at room temperature in a humid chamber with anti-dog polyclonal antisera for the detection of IgM, IgA, IgG, C3 (Bethyl Laboratories, Montgomery, Texas, USA), or fibrinogen (Bioclin, Belo Horizonte, Minas Gerais, Brazil), the sections were subsequently conjugated with fluorescein isothiocyanate. The slides were mounted with buffered glycerin at pH 8.5 and examined under a universal microscope equipped with a system for immunofluorescence microscopy.

For TEM, the samples were post-fixed in 1% osmium tetroxide in 0.2 M sodium cacodylate buffer. After inclusion in resin, semi-finished, toluidine blue-stained sections were prepared for evaluation and selection of glomeruli.

Reading of the slides by standard LM, fluorescence microscopy, and TEM was performed by a blinded nephropathologist, who analyzed the specimens according to criteria established by the World Health Organization. All animals had more than 20 glomeruli evaluated, following the guidelines for glomerulopathies. In specimens evaluated by immunofluorescence, deposits were classified as granular or linear, and information on their microanatomical location (mesangium, basement membranes, tubules, and/or blood vessels), distribution (focal, diffuse, segmental, or generalized), and fluorescence intensity (0, traits +1, +2, or +3 in dark field) was retrieved.

Descriptive statistics, as well as frequency distribution variables, were used for each group. Analysis of variance or the Student's *t*-test were used for parametric variables and the Mann–Whitney test for non-parametric variables. *p*-values of < 0.05 were considered significant. Calculations were performed using GraphPad Prism 7.0 software (San Diego, CA, USA).

## Results

### Clinical Aspects

The main clinical signs and changes observed in the physical examination in dogs with distemper (*n*= 13) were: oculonasal secretion (*n*= 12; 92%), hyporexia (*n*= 11; 84%), paresis of pelvic limbs followed by paresis of the thoracic limbs (*n*= 8; 61%), ataxia (*n*= 7; 53%), cough (*n*= 5; 38.8%), hyperkeratosis of the foot pads (*n*= 5; 38.8%), absence of proprioception (*n*= 4; 30.7%), vocalization (*n*= 4; 30.7%), mild to moderate dehydration (*n*= 4, 30.7%), seizures (*n*= 3; 23%), and nystagmus (*n*= 2; 15%).

### Hematological and Biochemical Variables Analysis

The laboratory findings of the 13 animals with distemper were as follows: lymphopenia (*n*= 10; 76.9%), leukocytosis (*n*= 6; 46.2%), eosinophilia (*n*= 6; 46.2%), thrombocytopenia (*n*= 5; 38.5%), moderate anemia (*n*= 5; 38.5%), mild anemia (*n*= 5; 38.5%), and thrombocytosis (*n*= 4; 30.8%).

Alterations observed in the serum as assessed by biochemical tests included hypoalbuminemia (*n*= 10; 76.9%) and hyperproteinemia (*n*= 3; 23.1%), which indicated an increase in circulating globulins (Table 1).

**Table 1:** Results of complete blood count and serum biochemistry tests of 10 healthy dogs (control group) and 13 dogs with distemper (Franca/SP).

**Variables**	**Groups**		***p*** **-value**
	**Control**	**Distemper**	
Blood cells ( × 10^6^/μL)^*^	6.92 ± 0.82	4.34 ± 1.20	<0.0001
Hemoglobin (g/dL)^*^	16.3 ± 1.83	9.82 ± 2.55	<0.0001
Hematocrit (%)^*^	47.88 ± 5.03	29.60 ± 8.01	<0.0001
Total leukocytes ( × 10^3^/μL)^*^	10.140 ± 1.845	8.615 ± 9.291.47	0.0101
Platelets ( × 10^3^/μL)	301.500 ± 47.7	332.384 ± 259.2	0.7150
Total protein (g/L)	5.6 ± 0.53	6.2 ± 1.2	0.1705
Albumin (g/L)^*^	3.24 ± 0.37	2.5 ± 0.6	0.0025
Creatinine (mg/dL)^†^^*^	1.1 (0.7–1.5)	0.7 (0.4–4.8)	0.0026
Urea (mg/dL)^†^	34.0 (14.0–60.0)	30.5 (16.0–355.0)	0.9876

† *Non-parametric variables. Values followed by an asterisk*

(^*^)*differed significantly between the groups analyzed (p < 0.05)*.

### Serum Protein Fractions

The animals with distemper exhibited a higher globulin concentration than those in the control group (*p*= 0.0319). The main globulins whose concentration was increased were alpha-1-antitrypsin (*p*= 0.0185), haptoglobin (*p*= 0.0017), and—among immunoglobulins—IgA (*p*= 0.0002) and heavy chain IgG (*p*= 0.0402). Levels of other proteins, such as ceruloplasmin, transferrin, acid alpha-1-glycoprotein, 44- and 23-kDa proteins, and light chain IgG were not statistically different between the distemper and control groups (Table 2). Proteins with a molecular weight of 44 kDa were not identified in the distemper group.

**Table 2:** Mean values and standard deviation of parametric variables, and median values with minimum and maximum values of serum electrophoretic variables in the control and distemper groups.

	**Proteins (mg/dL)**		***p*** **-value**
	**Control**	**Distemper**	
Total protein	6.2 ± 0.75	5.9 ± 1.2	0.4677
Albumin	3701.0 ± 557.0	3,347.0 ± 1,020.0	0.2013
Globulin^*^	3.14 ± 0.67	3.73 ± 0.92	0.0319
Ceruloplasmin^†^	29.0 (6.2 ± 47.9)	37.6 (7.9 ± 199.1)	0.1962
Transferrin^†^	185.1(0–540.0)	218.0 (27.2–917.0)	0.5973
Alpha-1-antitrypsin^*^	197.2 ± 32.4	296.7 ± 161.7	0.0185
Haptoglobin^†^^*^	18.6 (4.25–74.2)	66.0 (0–489.2)	0.0017
Alpha-1-glycoprotein acid^†^	48.36 (4.13–296.6)	44.5 (5.42–513.2)	0.9349
MW 23.000	396.1 ± 117.0	425.6 ± 171.8	0.5449
MW 44.000	14.71 ± 10.87	–	–
IgA^*^	14.61 ± 3.16	30.9 ± 15.43	0.0002
Light chain IgG^†^	248.2 (46.75–475.6)	226.6 (115.0–877.0)	0.4221
Heavy chain IgG^*^	613.4 ± 288.9	820.0 ± 280.4	0.0402

* *Significant statistical difference*.

† *Non-parametric data; MW, molecular weight*.

### Proteinuria

The UPC ratios (Table 3) were higher in the group of animals with distemper (0.98 ± 0.93) when compared to the control group (0.13 ± 0.06; *p* = 0.0091). None of the animals in the control group exhibited proteinuria; conversely, in the distemper group, eight animals (65.0%) exhibited significant proteinuria (UPC > 0.5). Hyperesthenuria was observed in six animals (*n* = 6; 46.1%), while a specific density <1.020 was observed in two animals (*n* = 2; 15.4%).

**Table 3:** Results of the urinary tests of 10 healthy dogs (control group) and 13 dogs with distemper. Franca/SP.

**Variables**	**Groups**		***p*** **-value**
	**Control**	**Distemper**	
Specific urinary density^†^	1.041 ± 0.01	1.039 ± 0.014	0.7714
UP/C^*^^†^	0.13 ± 0.06	0.98 ± 0.93	0.0091

† *Non-parametric variables*.

(*) *Values followed by an asterisk differed significantly between the groups analyzed (p < 0.05)*.

### Light Microscopy

**Figure 1:**
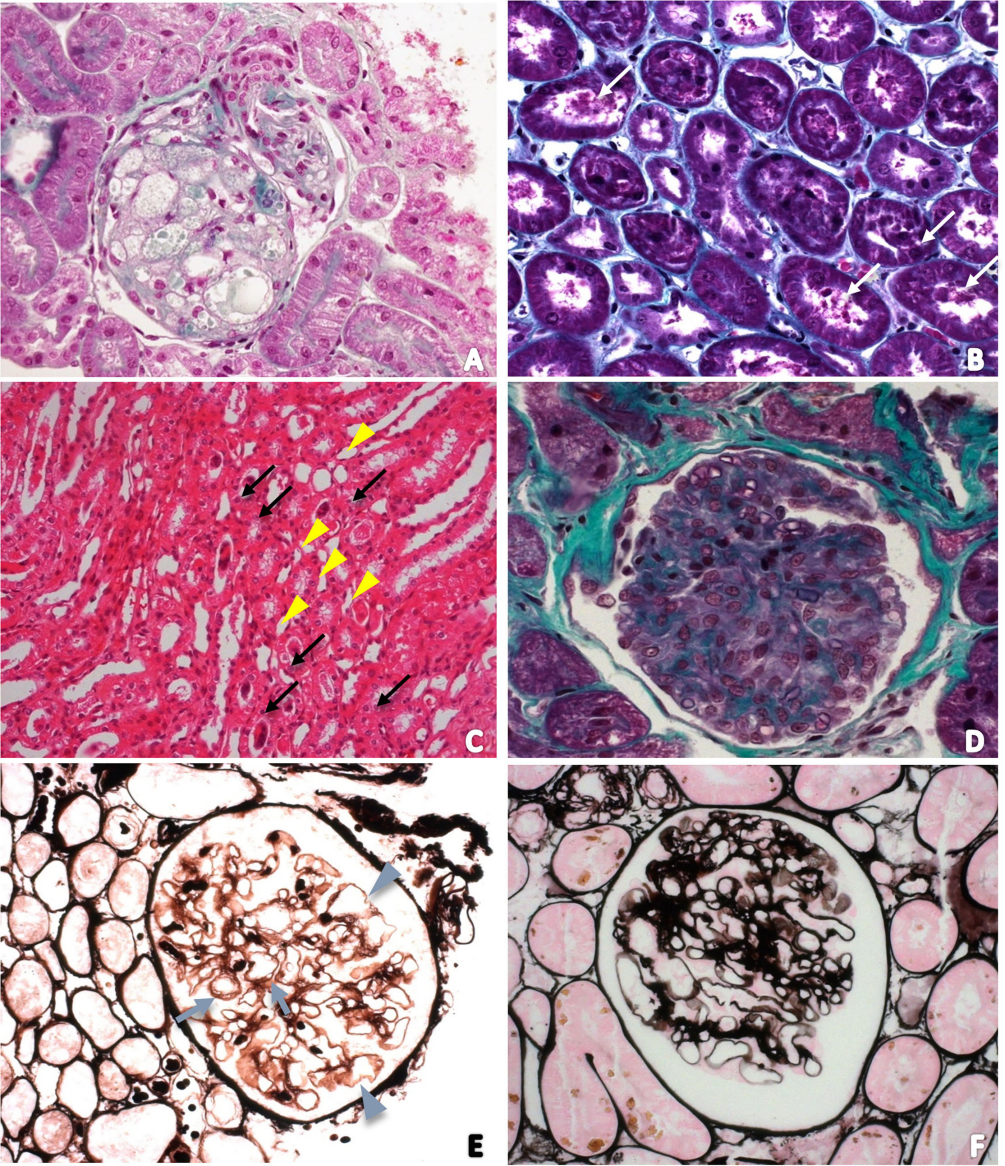
**(A)** Photomicrograph of a foamy glomerulus of a dog with distemper in a section stained with Masson's trichrome (400 ×); **(B)** Tubular necrosis in a section stained with Masson's trichrome (200 ×). White arrows indicate tubular cell detachment; **(C)** Photomicrograph of the renal medulla to characterize acute tubular necrosis. Tubular lumens are dilated and filled with necrotic cells (arrows). Some tubules have vacuolar degeneration (arrowhead). Hematoxylin and Eosin (100 ×); **(D)** Photomicrograph of glomerular congestion and endocapillary hypercellularity. There is increased numbers of intracapillary cells causing narrowing of glomerular capillary lumina; **(E)** Photomicrograph of ischemic changes in the glomerulus revealed by PAMS staining. Glomerular basement membrane duplication (arrows) and also wrinkling and irregularity of the loop (arrowhead); **(F)** Photomicrograph of the increased thickness of glomerular basement membranes without spikes and generalized expansion of the mesangial matrix revealed by PAMS staining.

Light microscopy evaluation did not reveal any alterations in specimens from animals of the control group. Renal changes were identified in 11 dogs (84.6%) of the distemper group. Among these changes, tubular alterations such as hydropic degeneration (*n* = 2; 15.4%—Figure 1B) and tubular necrosis (*n* = 3; 23.1%—Figure 1C) were observed. The glomeruli exhibited synechiae, perihilar focal segmental glomerulosclerosis (*n* = 3; 23.1%), glomerular congestion (*n* = 2; 15.4%—Figure 1D), ischemic changes in the glomerulus (*n* = 1; 7.7%—Figure 1E), necrosis and apoptosis (*n* = 1; 7.7%), generalized expansion of the mesangial matrix with thickening of the loops (*n* = 1; 7.7%—Figure 1F), increased thickness of glomerular basement membranes without spikes revealed by PAMS staining (*n* = 1; 7.7%—Figure 1F), fetal glomeruli with “crowns” of podocytes (*n* = 1; 7.6%), and presence of endocapillary foam cells (*n* = 1; 7.6%) (Figure 1A). Three animals (23.1%) exhibited globally sclerosed glomeruli. In the interstitium there was a mild deposition of fibrosis (*n* = 5; 38.5%) and a slight increase in cellularity (*n* = 3; 23.1%).

### Video Image Analysis for Assessment of Nuclear Phenotypes in RTECs

Nuclei in RTECs from the distemper group presented reduced values of area and perimeter compared to nuclei in RTECs from the control group (Table 4, *p* < 0.01). The RTECs from the distemper group presented condensed chromatin (high values of OD), which resulted in an increase in the textural contrast between nuclear areas of more and less condensed chromatin (Table 4). The mean IOD values (± standard deviation) related to the DNA content were 45.29 ± 4.22 AU for distemper group and 31.53 ± 5.17 AU for control group (*p* < 0.01).

**Table 4:** Mean values and standard deviation of the video image analysis parameters used to describe the nuclear phenotypes of the renal tubular epithelial cells.

**Parameters**	**Groups**		***p*** **-value**
	**Control**	**Distemper**	
Nuclear area (μm^2^)	68.37 ± 9.12	42.65 ± 11.56	<0.01
Nuclear perimeter (μm)	42.37 ± 5.84	32.14 ± 7.21	<0.01
OD (AU)	0.71 ± 0.14	0.89 ± 0.16	<0.01
SDtd	2.03 ± 0.42	2.78 ± 0.53	<0.01
IOD	31.53 ± 5.17	45.29 ± 4.22	<0.01

*AU, arbitrary units; OD, optical density; SDtd, standard deviation of the average pixel values per nucleus; IOD, integrated optical density*.

**Figure 2:**
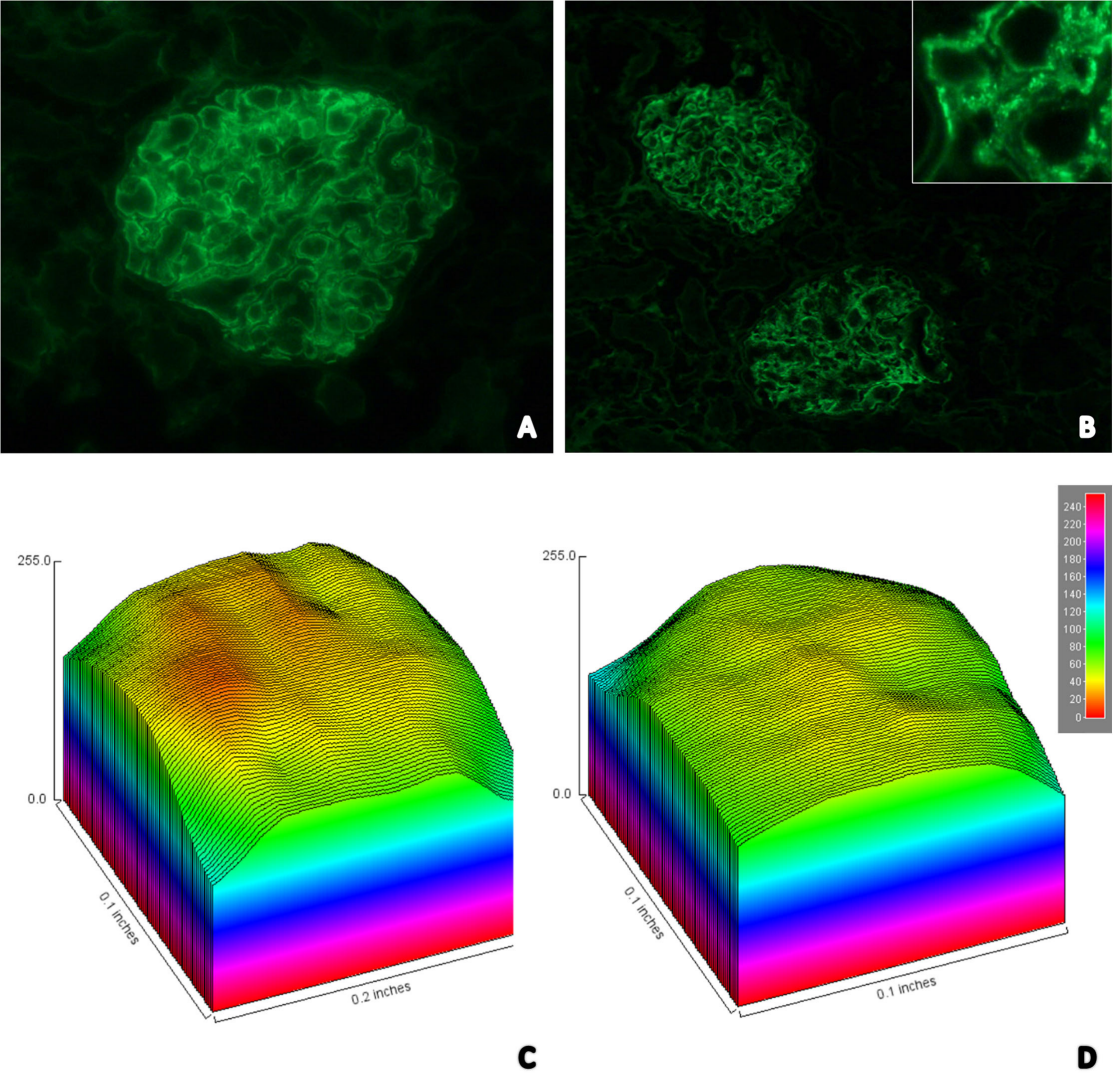
**(A)** Immunofluorescence image showing strong staining for glomerular IgM in a granular pattern in the mesangium; **(B)** Immunofluorescence image showing moderate staining for glomerular C3 in a granular pattern in the mesangium—In detail it is possible to identify the granular pattern (top right); Surface plot graph charts representative of the degree of condensation of chromatin presented by the RTECs in control **(C)** and distemper **(D)** groups. The nuclei contain 2 sets of chromatin with different states of supraorganization. The first set (green pseudocolor) had unpacking chromatin (41–60 pixels); and the second (orange) had highly compacted chromatin (20–40 pixels).

Details related to the compaction state of chromatin could be observed after the attribution of pseudocolors to nuclei and the construction of surface plot charts (Figures 2C,D). Nuclei in RTECs contain two sets of supraorganized chromatin, represented by different bitmap pixel values. The first set (green pseudocolor) had unpacking chromatin characterized by values between 41 and 60 pixels; and, the second (orange) had highly compacted chromatin, between 20 and 40 pixels.

### Immunofluorescence

Twelve (92.3%) of the 13 samples from the distemper group were positive for IgM (Figure 2A). Of these animals, two (16.7%) were positive only for IgM, four (33.3%) for IgM/C3 (Figure 2B), one (8.3%) for IgM/IgA, three for IgM/IgA/C3, one for IgM/IgG, one (8.3%) for IgM/IgG/IgA, and nine (69.2%) of the animals exhibited fibrinogen deposition in the glomerular loop with mesangial extension (Table 5).

**Table 5:** Renal positivity for IgG, IgM, IgA, C3, and fibrinogen assessed by immunofluorescence.

	**Control (*****n*** **=** **10)**		**Distemper (*****n*** **=** **13)**		***p*** **-value**
	**Positive IF**	**Median Intensity (min–max)**	**Positive IF**	**Median Intensity (min–max)**	
IgM	0	0 (0–0)	12	2 (0–3)	<0.0001
IgG	0	0 (0–0)	1	2	0.125
IgA	0	0 (0–0)	5	0 (0–2)	0.015
C3	0	0 (0–0)	7	0 (0–2)	0.062
Fibrinogen	0	0 (0–0)	9		

IgM deposits exhibiting moderate to high immunostaining intensity were observed in the mesangium and in the glomerular loop in 53.5% of the animals with distemper. C3 was detected in the glomerular membrane in 15.38% and in the glomerular loop in 15.38% of the cases. The intensity of immunostaining for IgA was high in the glomerular loop and in the mesangium in 15.38% of the animals.

### Transmission Electron Microscopy

**Figure 3:**
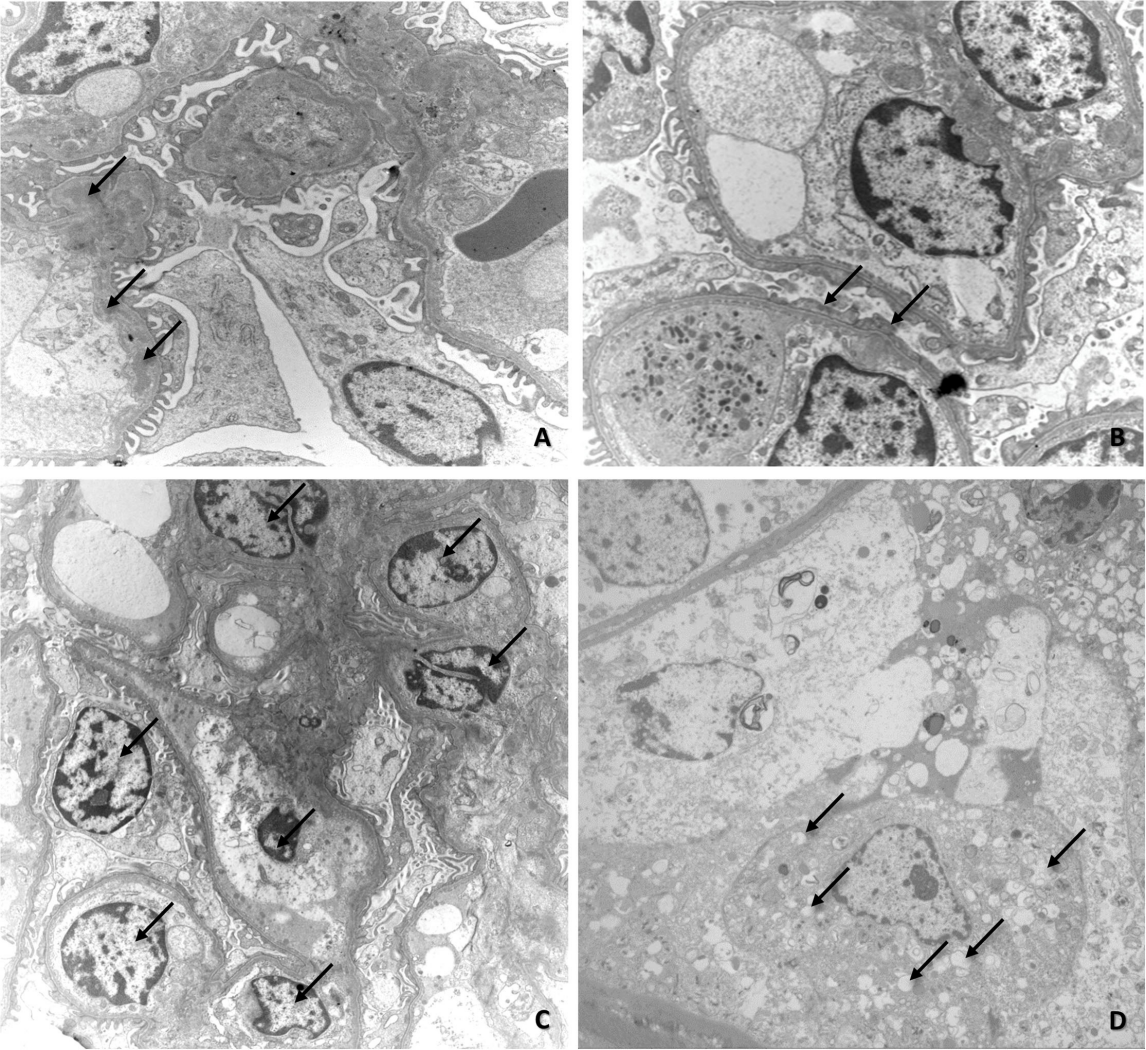
**(A)** Electron microscopy image of renal fragments exhibiting mesangial deposits of electron-dense material (arrows); **(B)** Electron microscopy image of renal fragments showing fusion of pedicels (arrows); **(C)** Electron microscopy image of increased mesangial cellularity (arrows); **(D)** Electron microscopy image showing initial stage of vacuolar degeneration in tubular epithelial cells (arrows).

Electron microscopy revealed the presence of deposits of electron-dense material in the subendothelial region of the mesangium (Figure 3A). Sometimes, the electron-dense deposits extended from the mesangium to the capillary loops, as previously observed by immunofluorescence. There were also discrete areas of glomerular basement membrane duplication, collagen fiber deposition, increased mesangial cellularity (Figure 3C), microvillous transformation (cytoplasmic microvilli-like projections into the urinary space that emanated from the luminal side of the podocyte membrane), congestion of the capillary loop, and focal fusion of pedicels (Figure 3B), and tubular alterations (tubular necrosis and hydropic degeneration—Figure 3D).

## Discussion

The results of this work unveiled the presence of renal alterations related to the canine distemper virus. Although many organs have been shown to be affected by the distemper virus (13), including those of the renal system (12), the tubular, and glomerular alterations are seldom described.

Creatinine in the distemper group was lower compared to the control group (*p* = 0.0026), but there was no significant difference in relation to urea (*p* = 0.9876). Although this alteration may be due to the smaller muscle mass observed in animals with distemper, it is probably not of clinical importance.

The UPC ratio was significantly higher in the group of animals with distemper when compared to the control group. Most of the animals of the group with distemper had significant proteinuria (UPC > 0.5), indicative of probable renal lesion (7). This inference was confirmed by the histopathological alterations found, which showed that both the glomeruli and the tubules were affected. Likewise, the protein fractions found in the urine of the animals affected by distemper were more pronounced in low-, medium-, and high-density fractions, indicating both glomerular and tubular involvement. Low-MW proteins (10–20 and 31–40 KDa) were found in cases of tubular proteinuria.

The decrease in protein intake consequent on the intestinal involvement owing to epithelial lesions caused by the virus was the determining factor for the reduction in serum albumin levels in the animals with distemper (14). This change was accompanied by an increase in circulating globulins, an alteration frequent in several inflammatory conditions, and which is significant particularly during bacterial and viral infections (14, 16). Proteinuria in these cases would make only a small contribution to the decrease in albumin levels, since it is reported that hypoalbuminemia is more related to marked proteinuria (UPC > 3.5) (17).

Viruses of the genus *Morbillivirus* cause serious diseases in animals and humans (18), and they have been reported as causative agents of renal damage (19–22), as seen in the experiment described herein. In this study, the most frequent changes found were interstitial nephritis and acute tubular necrosis, which were also described in studies related to measles (7, 8) and feline morbillivirus (9) and, to a lesser extent, hydropic degeneration. It is probable that there is also a component related to patient hydration, even though no compatible clinical changes were observed.

Similar changes in tubules and epithelia of the renal pelvis have been noted in cases of infection by the small ruminant plague virus, which exhibits a degree of neurovirulence very similar to that of the canine distemper virus, and this close viral relationship is highlighted by many authors (20). This neurovirulence results in high mortality, especially among young animals.

Kidney necrosis, such as that found in one of the animals with distemper in this study, has also been reported in horses as well as in humans diagnosed with a new type of morbillivirus (22) that manifests with clinical signs similar to those caused by the distemper virus, with a high mortality rate, which suggests that this results from a change in the virus–host interaction, confirming the introduction of the virus to new species.

Endothelial hypercellularity, mesangial matrix enlargement, and thickening of the glomerular capillary wall observed by LM and TEM in this study corroborate the pattern of glomerular lesion, which characterizes membranoproliferative glomerulonephritis patterns. In addition, nuclear phenotype patterns described by video image analysis of Feulgen-stained samples, which is the gold standard technique for chromatin-DNA studies (23), indicated a reduction in the metabolism of the RTECs of the distemper group. The increase in OD values found in the distemper group compared to the control group, reflecting an increase in the state of chromatin compaction, is indicative of a reduction in cell transcriptional activity. The changes found in OD, SDtd and IOD values, in association with a reduction in area and perimeter, are typical of nuclei in chromatin fragmentation. The nuclear alterations described for the RTECs in present study are similar to those described for the thymus (24) and cerebellum (15) of dogs infected with distemper, suggesting that chromatin fragmentation due to cell death or low physico-chemical stability of DNA is an event closely associated with the tissue lesions that accompany the disease.

Other important alterations, such as synechiae-containing, perihilar focal segmental glomerulosclerosis, generalized expansion of the mesangial matrix with thickening of the loops, and the presence of endocapillary foam cells, may be explained by an altered immune status of the animals, as described in cases of immune-induced glomerulonephritis (25). These alterations and vascular injuries caused by the virus could also cause ischemic changes in the glomerulus, as observed in one case.

Immunofluorescence revealed that most of the animals with distemper exhibited granular deposition of IgG and/or IgM associated with the C3 complement protein in the capillary wall, while electron microscopy showed electron-dense mesangial deposits; these findings are indicative of immune complex deposition. Immune complexes that attach to the glomeruli as a result of antigen entrapment lead to complement fixation and consequent glomerular lesions (25). Those complexes apparently have an important contribution to the genesis of renal damage in animals with distemper.

IgM is a large glycoprotein (880–941 kD) and may also be non-specifically trapped in glomeruli; passive capture may be responsible for some forms of glomerulonephritis associated with mesangial or subendothelial deposits (26). The presence of fibrinogen in the kidney specimens analyzed, especially in the glomerular loops and membranes, suggests that glomerulopathy also has an inflammatory and immune-mediated component. This assumption was confirmed by detection of increased levels of the acute-phase proteins haptoglobin and α1-trypsin.

Another important point that may be linked to the glomerulopathy is the increase in serum IgA and IgG levels, which may be related to the deposition of these immunoglobulins in the renal parenchyma and also perpetuation of the kidney damage observed by microscopy.

## Conclusion

In this study, the animals affected by distemper exhibited renal alterations related to the distemper virus at the tubular and glomerular levels. These alterations are due mainly to IgM-mediated immune complex deposition, with important tubular contribution.

## Data Availability Statement

The datasets presented in this study can be found in online repositories. The names of the repository/repositories and accession number(s) can be found in the article/supplementary material.

## Ethics Statement

The animal study was reviewed and approved by CEUA. Written informed consent was obtained from the owners for the participation of their animals in this study.

## Author Contributions

MS and LC designed the experiments and wrote the manuscript. MS, GS, MA, and CA performed the experiments. MS, SB-C, AA, LB, CP, and LC analyzed the data. AS, AA, SB-C, LB, CP, and LC reviewed the manuscript. All authors have read and agreed to the published version of the manuscript.

## Funding

This research was funded by Coordenação de Aperfeiçoamento de Pessoal de Nível Superior (CAPES—finance code 0001) and The São Paulo Research Foundation (FAPESP #2014/04743-0 and #2017/25458-0).

## Conflict of Interest

The authors declare that the research was conducted in the absence of any commercial or financial relationships that could be construed as a potential conflict of interest.

## Publisher's Note

All claims expressed in this article are solely those of the authors and do not necessarily represent those of their affiliated organizations, or those of the publisher, the editors and the reviewers. Any product that may be evaluated in this article, or claim that may be made by its manufacturer, is not guaranteed or endorsed by the publisher.
